# The effectiveness of computer reminders for improving quality assessment for point-of-care testing in general practice—a randomized controlled trial

**DOI:** 10.1186/1748-5908-8-47

**Published:** 2013-04-23

**Authors:** Marius Brostrøm Kousgaard, Volkert Siersma, Susanne Reventlow, Ruth Ertmann, Peter Felding, Frans Boch Waldorff

**Affiliations:** 1 The Research Unit for General Practice and Section of General Practice, Department of Public Health, Faculty of Health Sciences, University of Copenhagen, Øster Farimagsgade 5, P. O. Box 2099, Copenhagen DK-1014, Denmark; 2 Section of General Practice and Research Unit for General Practice, Department of Public Health, University of Copenhagen, Øster Farimagsgade 5, P. O. Box 2099, Copenhagen DK-1014, Denmark; 3 Copenhagen General Practitioners Laboratory, Pilestræde 65, Copenhagen K1112, Denmark

**Keywords:** Implementation, Point of care systems, Randomized controlled trial, Reminder systems, Quality assurance

## Abstract

**Background:**

Computer reminders are increasingly being applied in efforts to improve quality and patient safety. However, research is still needed to establish the effectiveness of different kinds of reminders in various settings. This study aimed to evaluate the effectiveness of computer reminders for improving adherence to a quality assessment scheme for point-of-care testing in general practice.

**Method:**

The study was conducted as a randomized controlled crossover trial among general practices in the Capital Region of Denmark. The intervention consisted of sending computer reminders (ComRem) to practices not adhering to the guideline recommendations of split testing for hemoglobin and glucose. Practices were randomly allocated into two groups. During the first follow-up period, one of the groups received the ComRem intervention together with the general implementation activities (GIA), while the other group only received the GIA. For the second follow-up period, the intervention was switched between the two groups. Outcomes were measured as split test procedure adherence.

**Results:**

A total of 142 practices were randomly allocated to the early intervention group and 144 practices to the late intervention group (the control group in the first follow-up period). In the first intervention period, the mean number of split tests performed in the group receiving ComRem group increased from 1.22 to 3.76 (out of eight possible tests) while the mean number of split tests increased from 1.11 to 2.35 in the group targeted by GIA only (p = 0.0059). After the crossover, a similar effect of reminders was observed. Furthermore, the developments in outcome measures over time showed a strong effect of computer reminders beyond the intervention periods.

**Conclusion:**

There was a significant effect of computer reminders on adherence to the quality assessment scheme for point-of-care testing. Thus, computer reminders seem to be useful for supporting the implementation of relatively simple procedures for quality and safety.

**Trial registration:**

ClinicalTrials.gov: http://NCT01152177

## Background

Point-of-care testing (POCT) is increasingly being employed in general practice to improve diagnostic capabilities and the delivery of timely patient care [1-4]. Since POCT results influence the daily decisions of GPs, it is clearly important that the results are accurate. Thus the quality of care will suffer from misleading POCT results; in worst case, inaccurate results may cause the general practitioner (GP) to overlook life-threatening conditions such as hypoglycemic incidences. In order to ensure the technical and professional quality of POCT in general practice, quality assessment schemes have been set up in several countries [5,6], and split testing has been found to be a cost-effective way of performing external quality assessment [7]. In the Capital Region of Denmark, external quality assessment is enforced through a split test procedure and an annual visit by a facilitator from the Copenhagen General Practitioners Laboratory (‘the Laboratory’). However, adherence to the monthly split test procedure has been low among the GPs in the Capital Region. Thus, previous to this study, one-half of the clinics did not perform the required split tests in the baseline period and no clinics performed more than three quarters of the possible number of tests (Table 1). Therefore, the Laboratory and the administration of the Capital Region planned to improve adherence by using computer reminders (ComRem) embedded in the GPs’ electronic medical records (in addition to the general activities to promote quality assessment). At the same time, the parties wanted to carry out a thorough evaluation of this new implementation method.

**Table 1:** Baseline data (January to April 2010): GP and practice characteristics and distribution of point-of-care tests in the two RCT groups

	**Total**	**Early ComRem**	**Late ComRem**
GP characteristics^b^	(n = 341)	(n = 170)	(n = 171)
Gender, n (%)			
Male	171 (50.2)	79 (46.5)	92 (53.8)
Female	170 (49.8)	91 (53.5)	79 (46.2)
Age, mean (SD)	56.3 (8.2)	55.8 (8.2)	56.8 (8.2)
Years as GP, mean (SD)	14.8 (9.6)	14.8 (9.4)	14.9 (9.8)
Practice characteristics	(n = 286)	(n = 142)	(n = 144)
Practice organization, n (%)			
Single handed	239 (83.6)	119 (83.8)	120 (83.3)
Group	47 (16.4)	23 (16.2)	24 (16.7)
Number of patients per doctor, mean (SD)	1,599 (404)	1,584 (395)	1,614 (413)
Performed point-of-care tests			
No. of Hemoglobin tests per practice, mean (SD)	39.8 (54.7)	41.5 (59.6)	38.0 (49.4)
No. of Glucose tests per practice, mean (SD)	47.2 (52.4)	52.1 (57.5)	42.3 (46.5)
Primary outcome			
No. of split tests (out of 8) per practice, mean (SD)	1.11 (1.37)	1.19 (1.44)	1.03 (1.29)
Secondary outcomes			
No. of practices with >0% of recommended split tests performed, n (%)	146 (51.1)	73 (51.4)	73 (50.7)
No. of practices with ≥75% of recommended split tests performed, n (%)	0 (0.0)	0 (0.0)	0 (0.0)

^a^P-value from a chi-squared test (categorical variables) or t-test (continuous variables).

^b^The tests on GP characteristics account for the clustering of GPs in practices.

Within the last ten years, several systematic reviews have assessed the effects of computer reminders in regards to changing provider behavior [8-11]. The most recent Cochrane review finds the effects of computer reminders to be small to moderate, but due to the variation between the different types of reminder interventions and their results, the review concludes that more ‘research is needed to identify what types of reminders work and when’ [11], p. 2. On this background, the purpose of this randomized controlled trial (RCT) was to assess the effectiveness of ComRem for improving adherence to the quality assessment scheme for POCTs.

## Methods

### Study participants

The study took place in the Capital Region of Denmark in 2010 and 2011. A detailed description of the study protocol has been presented elsewhere [12]. A total of 567 practices with a total of 739 GPs were eligible in the study area with a total population of 1.1 million. Only general practices conducting at least five POCTs for either hemoglobin or glucose—and practices that did not conduct POCT for International Normalised Ratio (INR)—during the baseline period were to be included in the study. The reason for excluding practices that conducted INR was that these practices were to be included in another RCT on the effect of computer reminders versus postal reminders [12]. The practices were identified via the database of the Laboratory and the GP-database of the Capital Region. Because the intervention was part of the formal implementation activities sanctioned by the Capital Region and the Laboratory, the sample in this study included all relevant practices irrespective of their wish to participate.

### The split test procedure

The quality assessment guidelines recommend a split test procedure in each practice each month for each POCT type. In the split test procedure, the POCT result is compared to the result of a blood sample (from the same patient) analyzed at the Laboratory. The quotient of these two results should ideally be 1.00, but if the quotient lays within the interval 0.84 to 1.16 for hemoglobin and 0.82 to 1.18 for glucose it is acceptable [13]. The result of the analysis is sent to the general practice for self-evaluation. If a practice does not respond to a case of unacceptable deviation by sending in a new split test, the Laboratory asks the practice to do so. If the result still deviates too much, the Laboratory contacts the practice to help them locate the problem.

### General implementation activities (GIA)

In 2010, the Laboratory generally stepped up its implementation activities concerning the external quality assessment of POCT in general practice. First, the importance of performing split tests was emphasized at the annual facilitator visit to each practice as well as in three laboratory newsletters. Second, a few technical changes were introduced that made it easier for the practices to order split tests (particularly, in April 2010, the ordering procedure was fully digitalized). During the intervention periods, the facilitators were not informed about the allocation status of the clinics prior to the visits.

### The intervention: computer reminders (ComRem)

In the Danish healthcare sector, a common standard for secure electronic communication, MedCom, between healthcare providers has been in use for a decade. Within MedCom, each general practice has been allocated to a unique location number and all electronic communication is fed into the GPs electronic patient journal system. When the electronic journal system is accessed by the GP (or appointed staff), the system shows all communication that must be opened and approved. Hence, the computer reminders in this study are neither postal reminders nor traditional e-mails.

**Figure 1:**
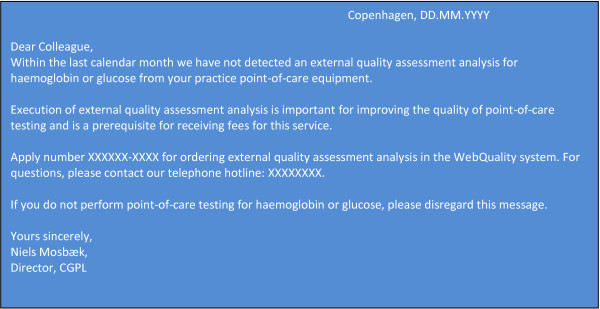
Content of computer reminder.

The intervention consisted of sending computer reminders (ComRem) to practices not adhering to the guideline recommendations of split testing for hemoglobin and glucose within the previous calendar month. Figure 1 shows the content of the reminders.

### Randomization and design

Practices were randomly allocated into two groups stratified by practice characteristics (group versus single handed) and geographic location. The randomization was done by means of computer-generated random numbers using SAS version 9.2. The randomization was performed by the data manager of the Research Unit of General Practice without knowledge of the individual practice identification. The allocation procedure was concealed from the project group and was conducted by an independent organization, the Danish College of General Practitioners. At enrolment, the project group delivered practice codes and information regarding stratification to the college where a designated staff conducted the allocation and returned the information to project group.

During the first follow-up period, one of the allocated groups (Early ComRem) received the ComRem intervention together with the GIA, while the other group (Late ComRem) received the GIA only. For the second follow-up period, the ComRem intervention was switched between the two groups so that all practices would receive ComRem in the overall study period. This crossover was chosen in order to accommodate our scientific interest in having a control group that did not receive the intervention with the wishes of the Laboratory and the Capital Region that all practices received the reminder intervention at some point during the study period in order to increase overall adherence. At the same time, the crossover design provided us with the opportunity to measure a legacy effect of the intervention.

### Sample size

To determine the power of the study, we used an estimate of a mean number of one hemoglobin and/or glucose split test based on laboratory data from 2007. Given a standard deviation of 1.25, a power of 90% and an effect of 0.5, we estimated that 266 practices were to be included in the study. We expected dropout rates to be negligible. Possible reasons for drop out were retirement or if a practice stopped using its own POCT equipment.

### Outcomes

Outcomes were measured as split test procedure adherence, *i.e.*, by the number of split tests received by the Laboratory. Outcomes were calculated and compared for three intervals of four months each:

1. The comparison of outcomes after the first intervention period estimates the relative effectiveness of ComRem versus GIA only.

2. The comparison after the second intervention period (in which the intervention was switched between groups) estimates the relative effectiveness of a (short-term) legacy effect of ComRem over the direct effect of ComRem.

3. The comparison after the third follow-up period (in which the ComRem intervention was discontinued for both groups) estimates the relative effectiveness of a longer-term legacy effect of ComRem over the short-term legacy effect of ComRem.

### Primary outcome

Total number of split test procedures for the corresponding POCT analysis performed by the practice in a four-month period. In a given month, a split test procedure should be performed if the practice conducted a POCT analysis for hemoglobin or glucose. Thus, the maximum number of possible split test procedures for a single analysis in a four-month period is four (*i.e.*, for both types of analysis the maximum number is eight). A reminder was only sent if both types of analysis are missing. Therefore, in each of the four months periods of this study, a practice could have received a maximum of four reminders.

### Secondary outcomes

1. Whether 75% of the required split tests were performed within the given follow-up period. This was defined as a high level of procedural quality assessment in the guidelines of the Laboratory.

2. Whether split test procedures were performed at all by the practice within the given follow-up period.

### Data collection

Data on the number of POCT tests conducted for each practice was retrieved from the Capital Region’s administrative database (in which information on the GPs reimbursement claims for POCT tests is stored). Data on the number of performed split test procedures were retrieved from the Laboratory database. This dataset does not contain information immediately relatable to specific patients because all split tests are performed with an artificial identification code. Data on the number of reminders were also obtained from the Laboratory. The Capital Region databases provided information on the participating practices and corresponding GPs.

### Statistical analysis

Differences in the GP characteristics and outcomes at baseline between allocation groups were tested by means of t-tests (continuous characteristics/outcomes) and chi-square tests (categorical characteristics/outcomes). Adherence to external quality assessment over time relative to the (changing) intervention, was analyzed by Poisson (primary outcome) and logistic (secondary outcomes) regression with GEE methods being used to account for the repeated measurements. SAS, version 9.2 (SAS Institute Inc, Cary, NC) was used for all statistical analyses.

## Results

**Figure 2:**
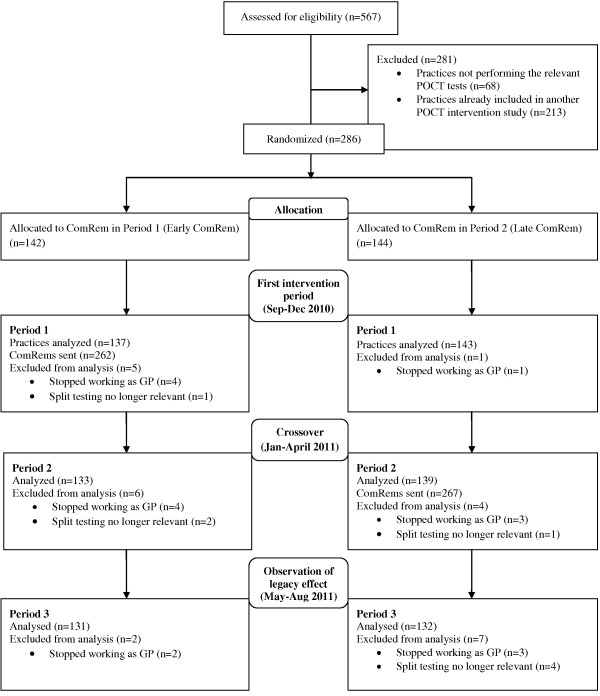
CONSORT flowchart.

A total of 286 general practices conducting at least five POCTs for hemoglobin or glucose during the four-month baseline period (1 January to 31 April 2010) were initially included in the study. In terms of GP and practice characteristics as well as the level of POCT-adherence, there were no significant differences between the two allocation groups (see Table 1). Figure 2 shows the flow of practices in the study. As expected dropout rates were very low. A total of 529 ComRems was sent to the practices during the two intervention periods.

### Outcomes

Table 2 shows the results for the main outcomes through the baseline period and three follow-up periods.

**Table 2:** **Main outcomes through the baseline period and three follow-up periods**^**a**^

	**Early ComRem**	**Late ComRem**	**p-value**^**b**^
**Baseline period**	**(n = 142)**	**(n = 144)**	
Intervention	General implementation activities (GIA)	General implementation activities (GIA)	
Primary outcome			
No. of split tests (out of 8) per practice, mean (95% CI)	1.22 (0.99 – 1.49)	1.11 (0.91 – 1.35)	0.5159
Secondary outcomes			
Practices with >0% of recommended split tests performed, fraction (95% CI)	0.51 (0.43 – 0.60)	0.51 (0.43 – 0.59)	0.9039
Practices with ≥75% of recommended split tests performed, fraction (95% CI)	0	0	–
**Period I** (First intervention period)	(n = 137)	(n = 143)	
Intervention	GIA plus ComRem	GIA	
Primary outcome			
No. of split tests (out of 8) per practice, mean (SD)	3.76 (3.39 – 4.16)	2.35 (1.99 – 2.78)	0.0059
Secondary outcomes			
Practices with >0% of recommended split tests performed, fraction (95% CI)	0.84 (0.77 – 0.89)	0.60 (0.52 – 0.68)	0.0000
Practices with ≥75% of recommended split tests performed, fraction (95% CI)	0.26 (0.19 – 0.34)	0.13 (0.08 – 0.19)	0.0065
**Period II** (Cross-over, second intervention period)	(n = 133)	(n = 139)	
Intervention	GIA	GIA plus ComRem	
Primary outcome			
No. of split tests (out of 8) per practice, mean (SD)	3.41 (2.96 – 3.94)	3.81 (3.41 – 4.26)	0.1030
Secondary outcomes			
Practices with >0% of recommended split tests performed, fraction (95% CI)	0.71 (0.63 – 0.78)	0.81 (0.74 – 0.87)	0.0406
Practices with ≥75% of recommended split tests performed, fraction (95% CI)	0.30 (0.23 – 0.38)	0.31 (0.24 – 0.40)	0.7614
**Period III** (Final follow-up after ComRem has been discontinued for both groups)	(n = 131)	(n = 132)	
Intervention	GIA	GIA	
Primary outcome			
No. of split tests (out of 8) per practice, mean (SD)	3.18 (2.75 – 3.66)	2.83 (2.43 – 3.31)	0.8800
Secondary outcomes			
Practices with >0% of recommended split tests performed, fraction (95% CI)	0.70 (0.62 – 0.77)	0.66 (0.57 – 0.73)	0.4707
Practices with ≥75% of recommended split tests performed, fraction (95% CI)	0.26 (0.19 – 0.34)	0.22 (0.16 – 0.30)	0.4345

^a^The values in the table are the predicted values derived from the Poisson regression model (primary outcome) or the logistic regression model (secondary outcomes) and their 95% confidence interval calculated with GEE methods.

^b^P-value pertaining to a test of difference between Early ComRem and Late ComRem (Baseline period) or a test of difference between Early ComRem and Late ComRem beyond the difference found in the baseline period (the three follow-up periods).

In the first intervention period, the mean number of split tests performed in the group receiving ComRem group increased by a factor of three from baseline to end of the first follow-up (1.22 to 3.76). In the other group targeted by GIA only, the primary outcome measure increased by factor of 2.12 (from 1.11 to 2.35). Compared to GIA without ComRem, GIA plus ComRem were significantly more effective in promoting adherence to guidelines; the number of split test performed was 1.40 (95% confidence interval: 1.07 – 1.83, p = 0.0059) times higher in the Early ComRem group than in the Late ComRem group beyond what could be expected by the difference between the groups at baseline.

For the secondary outcomes, the pattern was similar to the above in that 84% of practices in the Early ComRem group were performing at least one of the recommended split tests after the intervention against 51% at baseline and 60% in the late ComRem group.

In the second intervention period, in which the intervention was switched, split test adherence in the group now receiving ComRem increased to levels similar to those of the group that received ComRem in the first intervention period. Also, the results for the practices that received ComRem in the first period show a significant legacy effect of ComRem with adherence being close to that of the previous period.

In the third period, for which the ComRem intervention was discontinued for both groups, the effect of ComRem decreased for both groups, but adherence (in terms of the primary outcome) was still significantly higher for both groups compared to the baseline period.

## Discussion

### Main findings and interpretations

The results demonstrate a significant effect of computer reminders on all outcome measures. Furthermore, the developments in outcome measures during the various periods of the study show a strong legacy effect of computer reminders. However, while the computer reminders proved to be quite effective in nudging most practices towards a higher degree of adherence to quality assessment guidelines, the reminders were ineffective with regard to a minority of practices. Thus, 16% and 19%, respectively, of the practices were not performing any of the recommended split tests after each group had received the ComRem intervention. The increase in adherence to quality standards observed in the late ComRem group during the first period of the study may be ascribed to the general strengthening of implementation activities previously mentioned.

### Strengths and limitations

This study had several methodological strengths. First, the outcome measures were highly valid in being independent of subjective perceptions in the study population. Second, selection bias in the study population was avoided, because inclusion in the study was not based on voluntary participation. Third, dropout rates during the intervention period were very low. Furthermore, in this setting ComRem seems to be a feasible intervention, which can easily be converted into a permanent method for supporting implementation of external quality assessment. Thus, the reminders employed in this study constitute a relatively simple and low-cost technology compared to advanced computerized decision support systems. However, the technical infrastructure required for sending automatically generated reminders straight to the GPs electronic patient journal obviously puts certain limits on the immediate transferability of the intervention beyond this particular setting. Also, it could be argued that solely measuring process adherence represents a limitation because this means that we do not know whether the actual quality of the point-of-care tests improved along with the increased adherence to procedural standards. This points to another limitation, namely that the quantitative data does not tell us what specific actions are taken by practices who wish to step up adherence when they receive a reminder (*e.g.*, going over the correct use of the testing equipment, recalibrating the equipment, or changing roles and responsibilities in the practice).

### Comparison with literature

The most recent Cochrane review on computer reminders [11] found that reminders generally had little or moderate success in changing adherence to recommended processes of care (although a few studies in the review showed stronger effects). There may be at least two reasons why the effects of computer reminders in this study were far more pronounced than reported by the majority of studies in the Cochrane review:

1. While the Cochrane review focused on reminders appearing on the computer screen during the patient encounter with the objective of improving patient care processes and outcomes (*e.g.*, many of the studies centred on prescription) [11], the objective of reminders in this study was to change provider behaviour in regards to managing laboratory tests. Thus, this study deals with behavioural adherence with regard to a relatively simple procedure (split testing), and it is likely that the effect of ComRem will be reduced if employed to support the implementation of more complex recommendations [14].

2. Using electronic reminders to support quality assurance is a new phenomenon in Danish general practice. Hence, a certain ‘novelty effect’ may be present in this study. It is possible that the effect of electronic reminders may decrease if they become more widely used, giving rise to a situation in which several different reminders compete for the attention of health professionals.

It should be mentioned that the possible financial consequence of non-compliance stated in the reminder was probably not taken too seriously by the doctors because they have previously been informed about this and because most of them are probably aware that restriction of payment has not yet been applied in response to non-compliance.

## Conclusions

While previous studies have reported small to moderate effects of computer reminders, the results of this study suggest that electronic reminders can be a quite effective tool for changing professional behavior with regard to quality assurance of medical equipment. Future research could explore: how electronic reminders are perceived by health professionals, *e.g.*, whether and when they are perceived as helpful or disturbing; how the concurrent operation of different electronic reminders may affect the effectiveness of each other; and the specific circumstances that amplify or reduce the effectiveness of electronic reminders.

In terms of practical implications, the results from this study have encouraged the Laboratory to make computer reminders part of its routine implementation practice—a move that attests to the feasibility of computer reminders in this setting. Furthermore, in continuation of this study, the Laboratory may attempt to identify the technical and organizational changes employed by some of the practices that improved adherence during the intervention. Such knowledge could be used to assist other practices in adopting a more systematic approach to quality assurance for POCT.

Finally, and on a more general level, it should be noted that the increased technical possibilities of employing electronic reminders place a responsibility on policy makers and quality assurance actors to coordinate and prioritize the use of electronic reminders in order to avoid an overflow of reminders at the clinic level.

### Ethics

This study used blood samples that were not retraceable to specific patients in order to conduct the split tests. Patient identities were kept hidden through an artificial identification code used by all practices. The project has been evaluated and approved by the Danish Data Protection Agency (j. nr. 2010-41-4680) and the Danish College of General Practitioners Study Committee (MPU 12–2010). The study was also submitted for assessment by the Scientific Ethical Committee for Copenhagen and Frederiksberg Municipalities (j. nr. H-1-2010_FSP/10). The committee found that approval was not necessary since the study was not clinical.

## Abbreviations

ComRem: Computer reminders embedded in GPs electronic medical records; CONSORT: Consolidated Standards of Reporting Trials; GIA: General implementation activities; GP: General practitioner; The Laboratory: Copenhagen General Practitioners Laboratory; POCT: Point of care testing; RCT: Randomized controlled trial

## Competing interests

The study was initiated by FBW who is a GP in the study area. PF is working in the Copenhagen General Practitioners Laboratory that provided data for the study and sent out the reminders.

## Authors’ contributions

MBK participated in the design of the trial, and wrote the first manuscript draft. VS participated in the design of the trial, and did the statistical analysis. SR and RE participated in the design of the trial. PF participated in the design of the trial, provided data, and managed the reminder procedures. FBW initiated the trial, and participated in the design and management of the trial. All authors have commented on and approved the manuscript.
